# Supplementary figures and images for: Cellular morphology of leg musculature in the water bear Hypsibius exemplaris (Tardigrada) unravels serial homologies
Source: R Soc Open Sci. 2019 Oct 16;6(10):191159. doi: 10.1098/rsos.191159 (PMC6837179; doi:10.1098/rsos.191159)

Myosin blot Blotted gel

~250

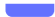

~130

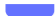

~100

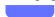

~70

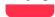

~55

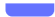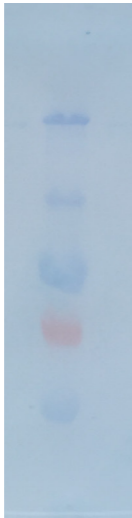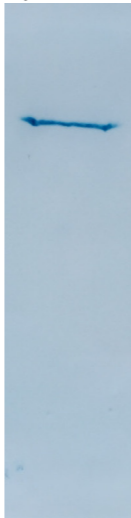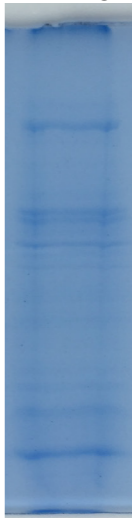

Supplement: Figure S1 [file rsos191159supp1.pdf]
