# Supplementary material for: Cellular morphology of leg musculature in the water bear Hypsibius exemplaris (Tardigrada) unravels serial homologies
Source: R Soc Open Sci. 2019 Oct 16;6(10):191159. doi: 10.1098/rsos.191159 (PMC6837179; doi:10.1098/rsos.191159)
Supplement: Table S1 [file rsos191159supp5.pdf]

Supplementary Table 1. Leg I. Comparison of leg muscle terminology from the literature and this study for *Hypsibius exemplaris* and closely related species. Note that labels from Müller [10] are compiled from several species. Labels are taken directly from references (from top to bottom): Müller [10] (*H. convergens, Ramazzottius oberhaueseri, Macrobiotus hufelandi*), Schmidt-Rhaesa & Kulessa [11] (*Hypsibius* sp.), Smith & Jockusch [15] (*H. exemplaris*), Marchioro *et al*. [14] (*Acutuncus antarcticus*), this study (*H. exemplaris*). Question marks indicate ambiguous or missing data. See main manuscript for references.

	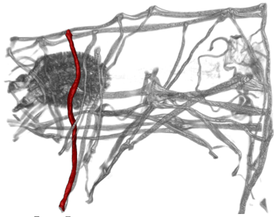	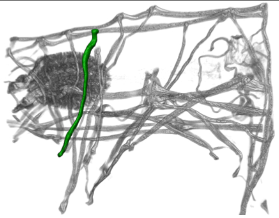	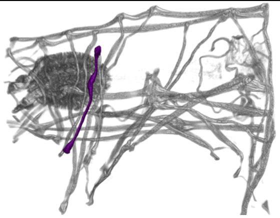	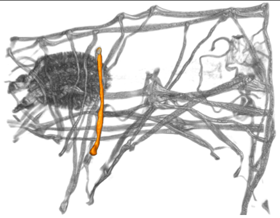	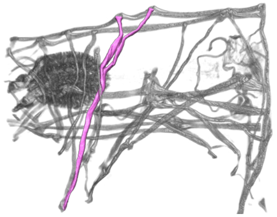	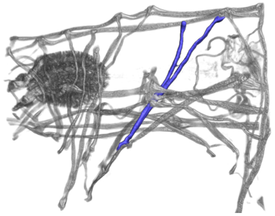
Ref. [10]	∂	3	ε	ε	4, 5	6, 7a
Ref. [11]	d1	e1	e2	e3	f1, g1	h1, i1
Ref. [15]	d1	e1	f*1	f*2	f1, g1	h1, i1
Ref. [14]	∂_1–LI2	2–LI5	ε–LI3	ε–LI3	5–LI1, 4–LI4	6–LIa, 7–LIa
This study	iM1	iM2	iM3	iM4	iM5	iM6
	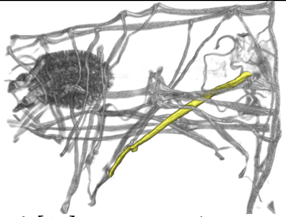	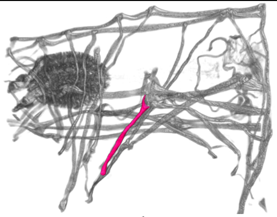	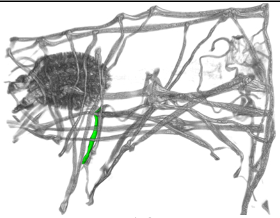	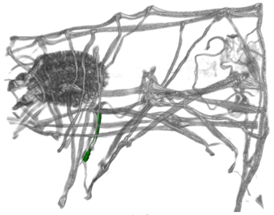	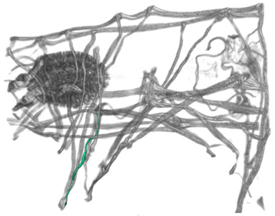	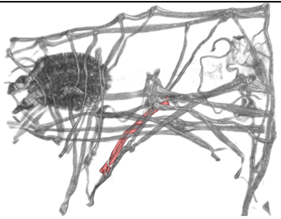
Ref. [10]	l	d	A?	A?	A	B
Ref. [11]	t1	τ1	1i-a	1i	?	2i
Ref. [15]	t1	τ1	1*	1i	?	2i
Ref. [14]	I–LIa + LIa–LIb	d–LIb	A–LIc	A–LIc	A–LId	B–LIb
This study	iM7	iM8	iM9	iM10	iM11	iM12
	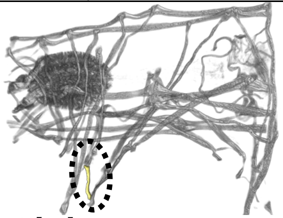	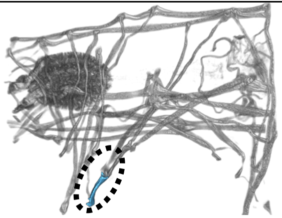				
Ref. [10]	?	?				
Ref. [11]	?	?				
Ref. [15]	?	sh				
Ref. [14]	?	LIb (proximal point)				
This study	iM13	iM14				

Table 1 (continued). Leg II.

	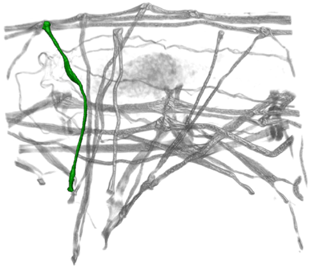	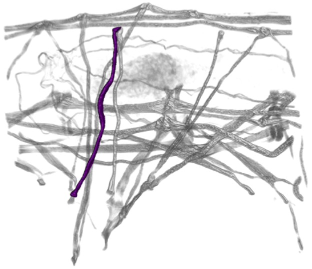	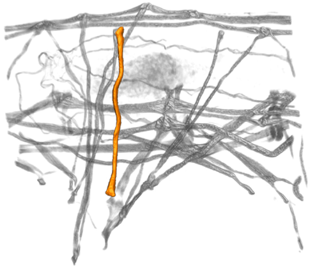	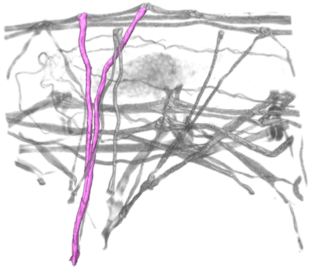	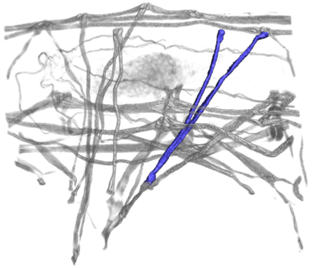	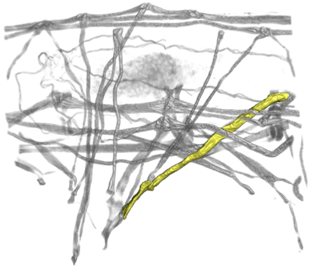
Ref. [10]	7a	9b	9a	7b, 9a	10, 11a	i
Ref. [11]	i2	l2	l3	k1/2, l1	n1, o1	t2
Ref. [15]	i2	l*1	l*2	k1, l1	n1, o1	t2
Ref. [14]	7–LII5	8–LII4	8–LII3	8–LII2, 9b–LII6	10–LIIa, 11–LIIa	i–LIIa + LIIa–LIIb
This study	iiM2	iiM3	iiM4	iiM5	iiM6	iiM7

	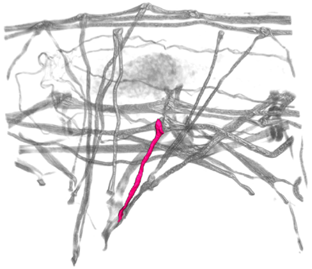	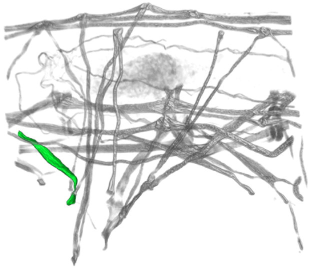	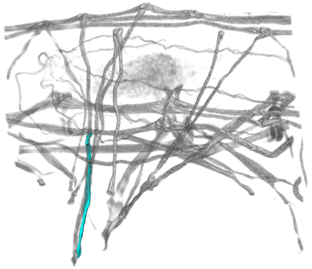	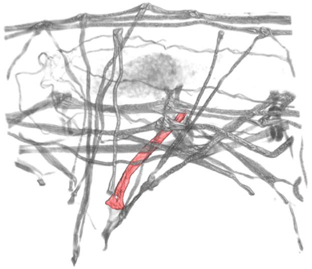	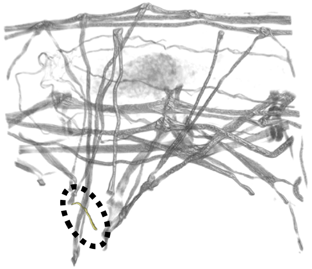	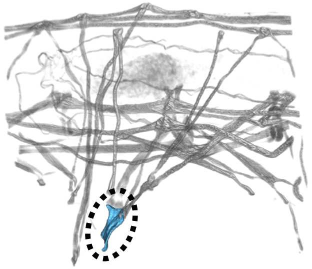
Ref. [10]	e	B	C	D	?	?
Ref. [11]	τ2	2iii	3i	4i	?	?
Ref. [15]	τ2	2iii	3i	4i	?	sh
Ref. [14]	e–LIIb	B–?	C–LIId	D–LIIb	?–LIIe	LIIb (proximal point)
This study	iiM8	iiM9	iiM11	iiM12	iiM13	iiM14

Table 1 (continued). Leg III.

	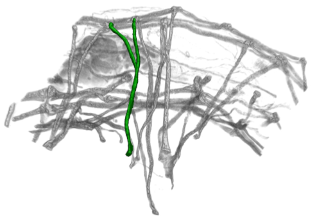	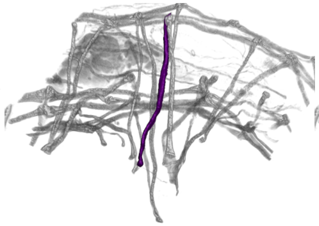	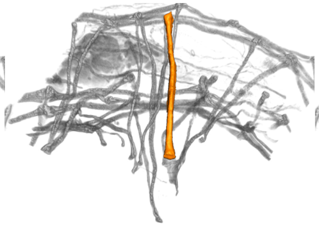	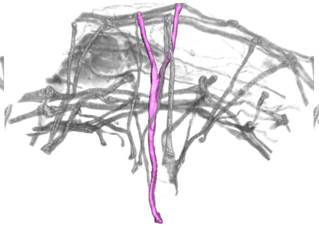	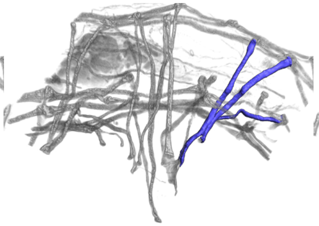	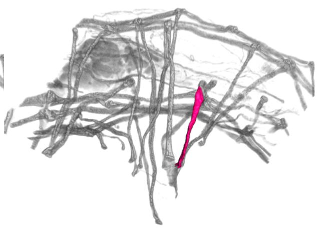
Ref. [10]	11a, 11b	13b	13a	11b, 13a	Λ, η, g_2	f
Ref. [11]	o2, P?	q2	q3	P?, q1	s1, u1, t3	τ3
Ref. [15]	o2, p2	q*1	q*2	q1	s1, u1, t3b	τ3
Ref. [14]	11–LIII2	13–LIII5	13–LIII3	12–LIII1, 13–LIII1	Λ, $η_a$, g_2–LIIa	f–LIIIb
This study	iiiM2	iiiM3	iiiM4	iiiM5	iiiM6	iiiM8
	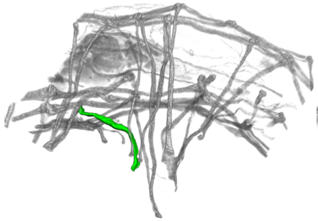	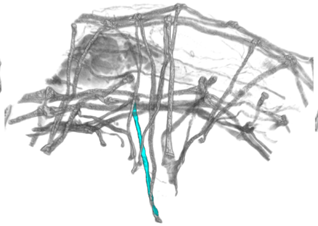	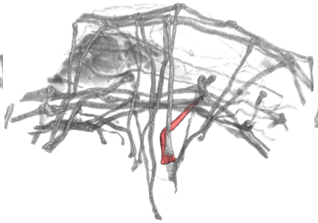	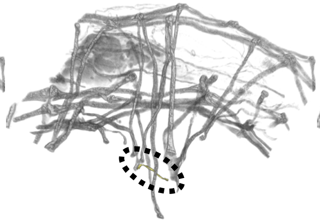	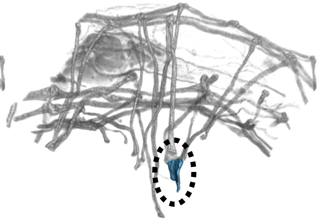	
Ref. [10]	D	E	F	?	?	
Ref. [11]	4iii	5i	6i	?	?	
Ref. [15]	4iii	5i	6i	?	sh	
Ref. [14]	D–?	E–LIIId	F–LIIIb	?–LIIIe	LIIIb (proximal point)	
This study	iiiM9	iiiM11	iiiM12	iiiM13	iiiM14	

Table 1 (continued). Leg IV.

	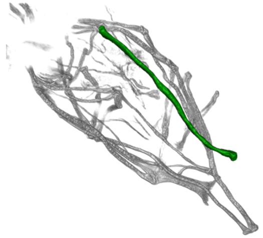	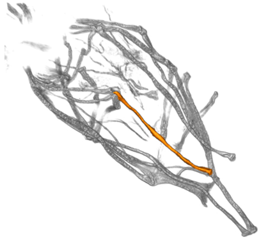	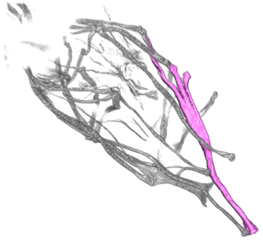	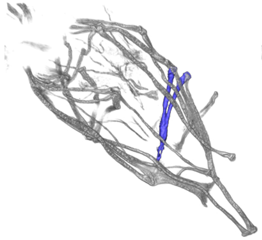	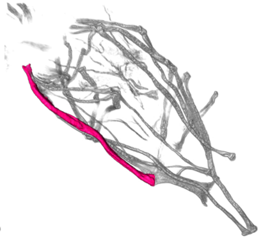	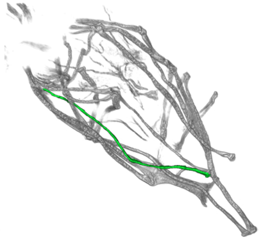	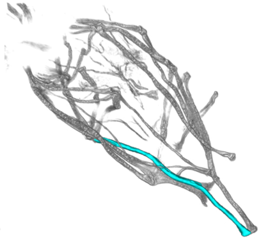
Ref. [10]	χ–η	ϕ–g_2	P_2–Q, μ, θ	?	O–f	ϕ–F	drawn, not labelled
Ref. [11]	u2	?	w’1, w1, v1	?	8a?	?	7iii
Ref. [15]	?	?	w1	?	8a	?	7iii
Ref. [14]	?	?–g_2	P_2–θ, χ	χ–O	O–f	χ–G	P_2–N
This study	ivM2	ivM4	ivM5	ivM6	ivM8	ivM9	ivM11

	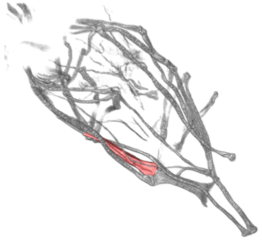	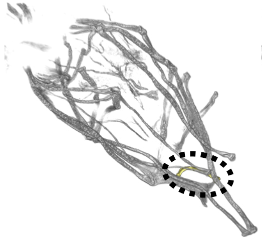	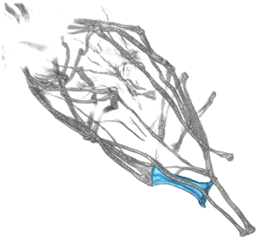
Ref. [10]	?	P_1–an	P_1–O
Ref. [11]	?	?	g
Ref. [15]	?	?	sh
Ref. [14]	O–N	?–P1	P1–O
This study	ivM12	ivM13	ivM14
